# Cancer History, Antiphospholipid Syndrome, and Lupus Anticoagulant: A Perfect Storm for Thrombosis

**DOI:** 10.7759/cureus.76481

**Published:** 2024-12-27

**Authors:** Sabrina Carpintieri, Elias Uyar, Christian Anand, Yaroslav Buryk

**Affiliations:** 1 Internal Medicine, Ross University School of Medicine, Miami, USA; 2 Internal Medicine, St. George's University School of Medicine, Brooklyn, USA; 3 Pulmonary and Critical Care, Jackson Memorial Hospital, Miami, USA

**Keywords:** antiphospholipid syndrome, cancer, cancer remission and thrombosis, complex anticoagulation management, hypercoagulable state, lupus, lupus anticoagulant, multidisciplinary management, thrombophilia, thrombotic disorders

## Abstract

Cancer and antiphospholipid syndrome (APS) independently increase thrombotic risk, and their coexistence can create a particularly hazardous prothrombotic state. This case report aims to highlight the complex challenges in managing concurrent thrombotic and hemorrhagic events in patients with a history of cancer and APS. The combination of these conditions presents a rare and difficult clinical scenario, requiring careful consideration in anticoagulation management. By presenting this case, we seek to emphasize the persistent thrombotic risk in cancer survivors and the importance of considering APS in patients with unexplained thrombotic events. This case report presents a 51-year-old male with a history of duodenal cancer in remission who experienced multiple severe thrombotic events, including an acute ischemic stroke, intracranial hemorrhages following treatment, and a pulmonary embolism. APS was diagnosed based on these events and positive laboratory findings. Management involved a delicate balance between thrombotic and hemorrhagic risks, with anticoagulation initially withheld due to intracranial hemorrhages, then cautiously initiated following the pulmonary embolism. The patient gradually improved, regaining functional independence with mild residual weakness three months post-discharge.

## Introduction

The association between malignancy and the development of blood clots is well-established. Studies have shown that approximately 20% of all venous thromboembolism (VTE) cases occur in cancer patients. Moreover, individuals with cancer face a four- to sevenfold higher risk of developing venous thrombotic events compared to those without cancer [1]. This hypercoagulable state seen in cancer patients is multifactorial, involving the release of procoagulant factors and inflammatory cytokines [2]. The combination of cancer-induced hypercoagulability and lupus anticoagulant creates a *perfect storm* for thrombotic events. 

Antiphospholipid syndrome (APS) is an acquired prothrombotic condition characterized by the presence of antiphospholipid antibodies (aPLs), anticardiolipin, and anti-β2 glycoprotein I antibodies [3]. This complex disorder can precipitate both venous and arterial thrombosis, potentially leading to life-threatening complications across multiple organ systems. 

The coexistence of both cancer and APS poses a unique challenge in patient management. While both conditions independently increase thrombotic risk, their combined effect can lead to a dramatically elevated risk of thrombotic events. Recent studies suggest an elevated prevalence of certain cancers in aPL-positive patients, particularly hematologic malignancies (notably non-Hodgkin's lymphoma) and certain solid tumors such as lung and breast cancer [4]. Conversely, elevated levels of aPL have been reported in various malignancies, although their pathological significance in cancer patients remains unclear. The aPL levels in cancer patients do not always correlate with their pathogenicity, complicating the interpretation of these findings.

## Case presentation

A 51-year-old male with a past medical history of duodenal cancer (in remission for four years), chronic back pain, and hyperlipidemia presented to the emergency department with a sudden onset of right-sided weakness and difficulty speaking. The patient's home medications included rosuvastatin (20 mg daily) for hyperlipidemia and aspirin (81 mg) for cardiovascular protection.

Upon initial examination, the patient was alert and oriented to person and place with prompting, but demonstrated right upper and lower extremity weakness (strength 0/5) and full strength (5/5) on the left side. An initial non-contrast brain CT was negative for acute changes. However, given the neurological deficits, a CT angiogram (CTA) of the head was performed, revealing an occlusion of the LP2 segment (left posterior cerebral artery, segment 2).

**Figure 1:**
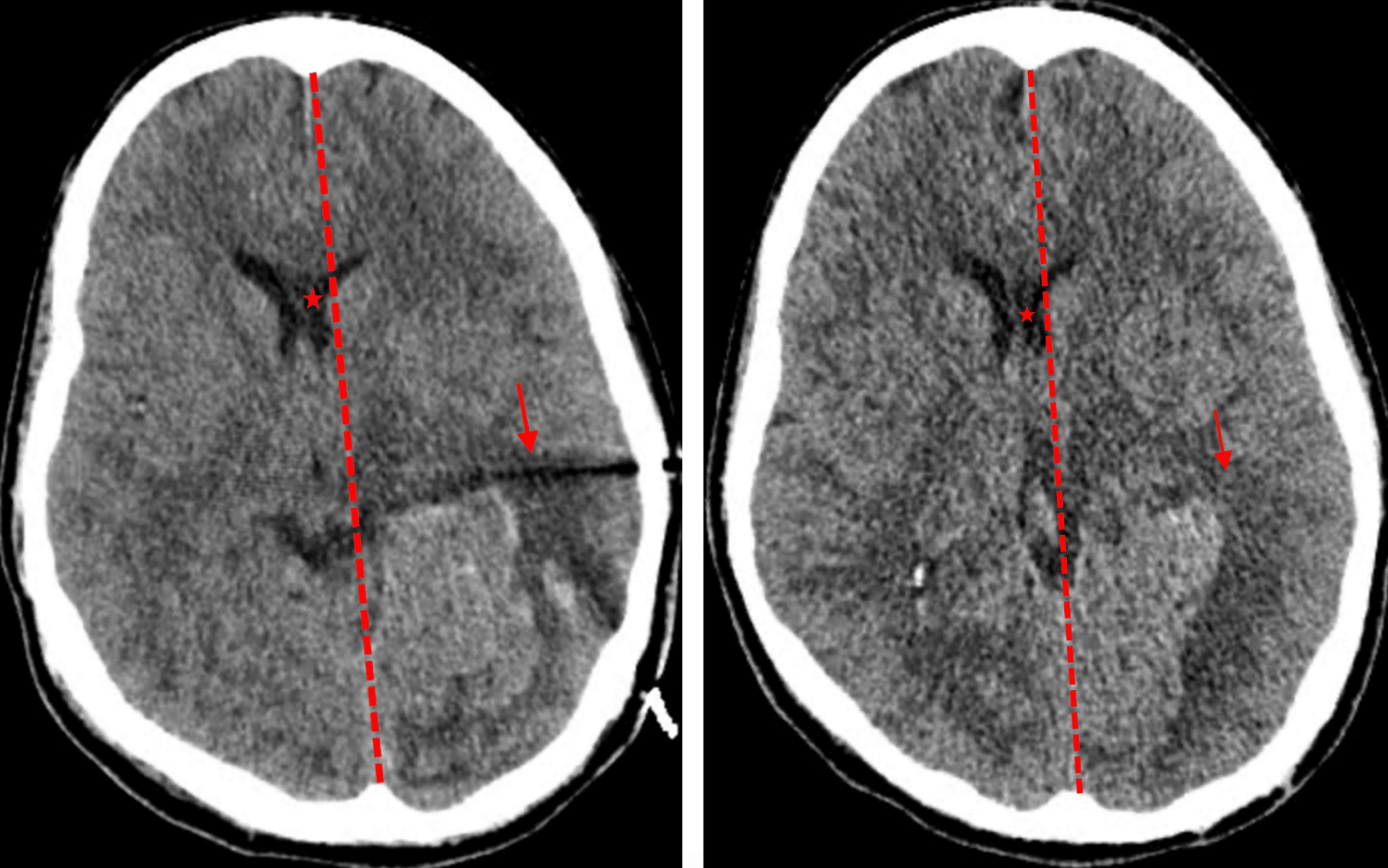
(A) Non-contrast brain CT; (B) subsequent non-contrast brain CT. The red arrow indicates a hypodense area in the left temporal lobe. The dashed red line indicates a midline shift toward the right. The red star depicts the rightward shift of the falx cerebri.

Based on these findings, the patient received tissue plasminogen activator (tPA) followed by mechanical thrombectomy and intra-arterial tPA infusion targeting the LP2 occlusion. Despite initial improvement, a follow-up brain CT on day 1 revealed two left posterior temporal lobe hemorrhages with intraventricular extension and subfalcine herniation to the right, which remained stable on subsequent imaging (Figures 1A-1B).

**Figure 2:**
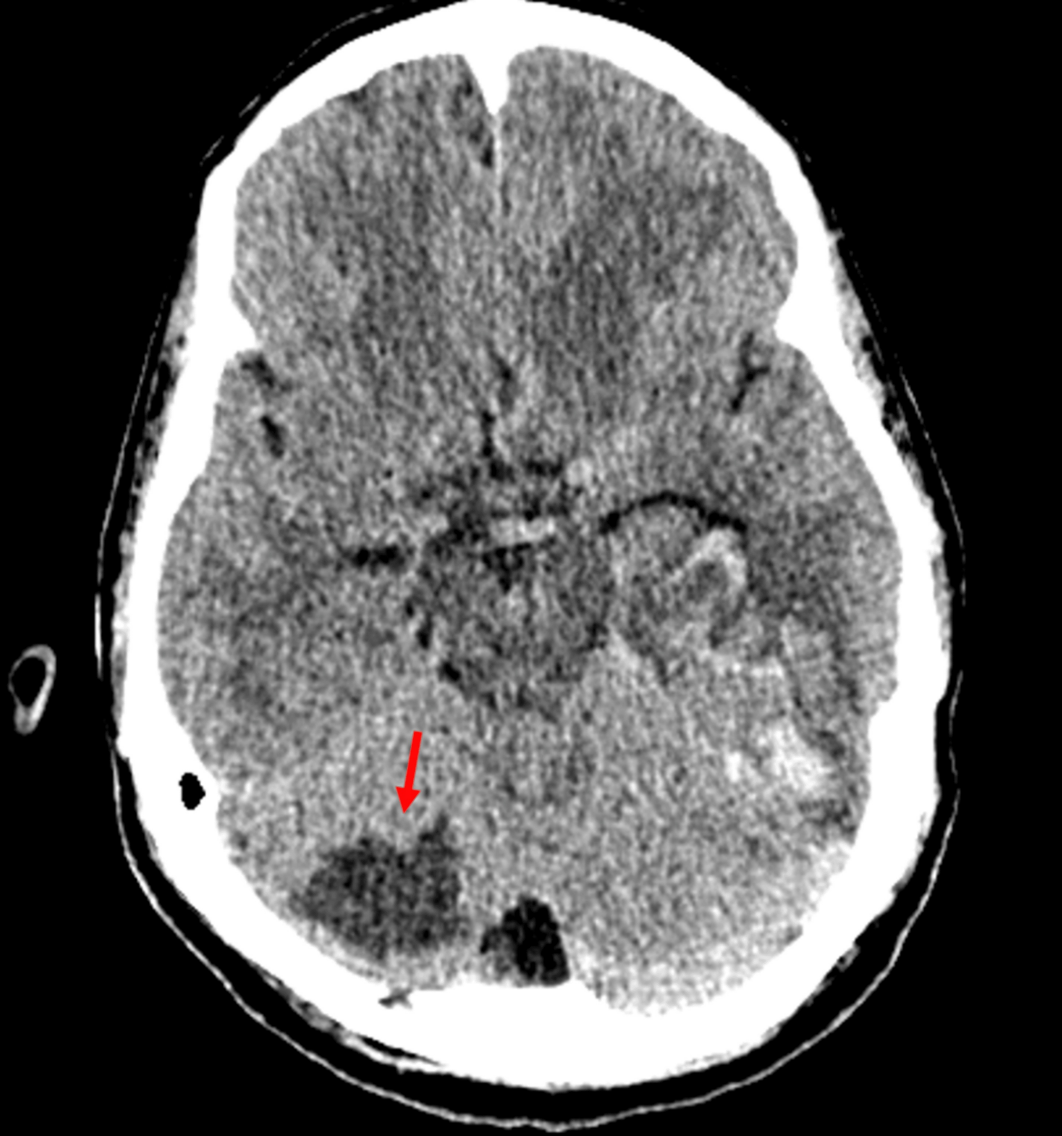
Non-contrast brain CT. The red arrow is pointing to the hypodense lesion.

Imaging revealed a circumscribed hypodense lesion in the superior cerebellum, suggestive of a cystic mass or sequelae of a prior infarct (Figure 2). 

**Figure 3:**
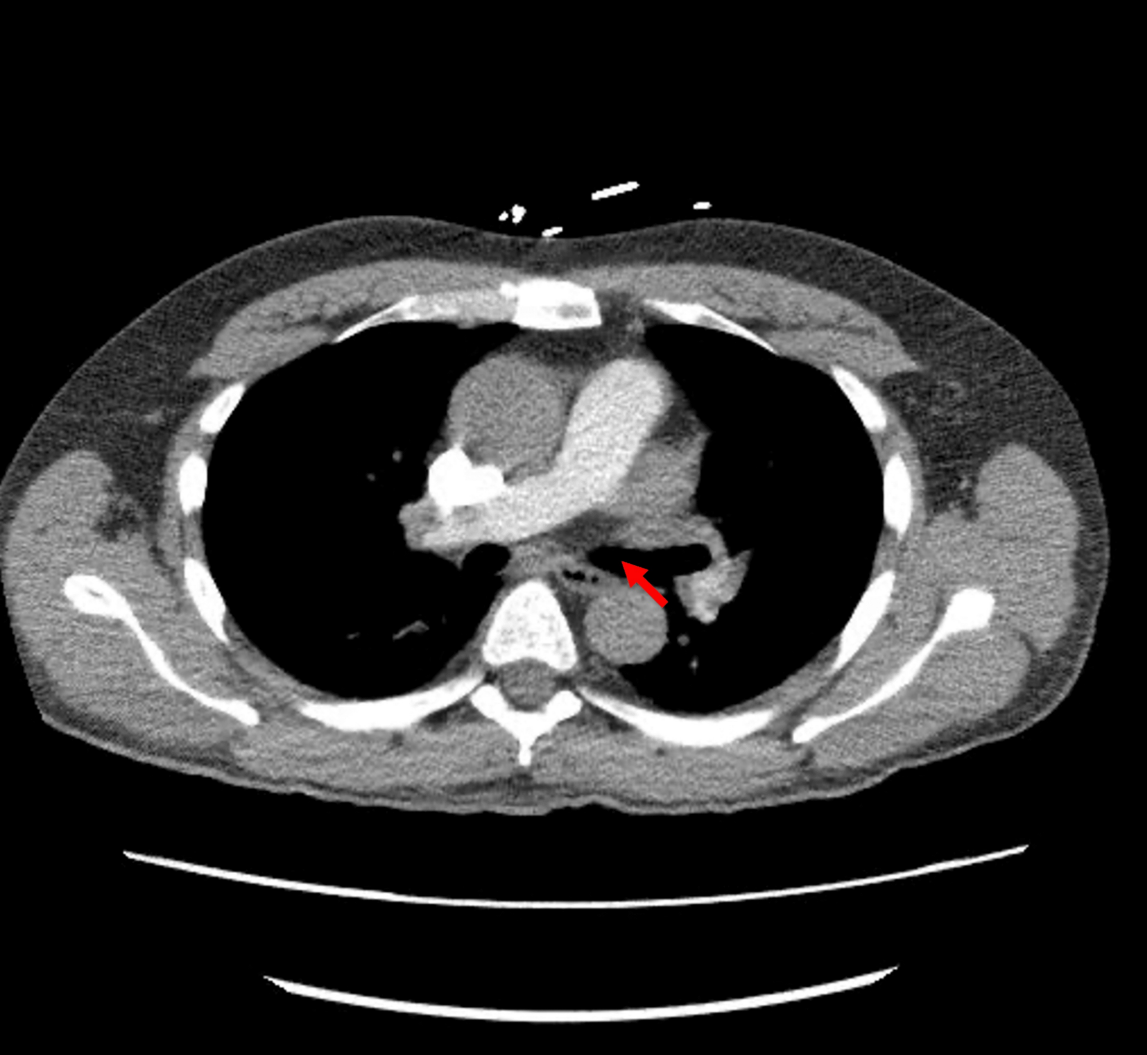
CT pulmonary angiogram demonstrating left lower lobe pulmonary embolism. This is an axial slice of a contrast-enhanced CT pulmonary angiogram at the level of the lower pulmonary arteries. The red arrow indicates a filling defect in the left lower lobe pulmonary artery, consistent with an acute pulmonary embolism. Note the central lucency within the contrast-opacified vessel, indicative of the embolus.

On day 2, the patient's condition worsened as he became acutely tachycardic and hypoxic. A CTA of the chest was performed, revealing a large pulmonary embolism (PE) in the artery to the left lower lobe basal segments (Figure 3).

**Figure 4:**
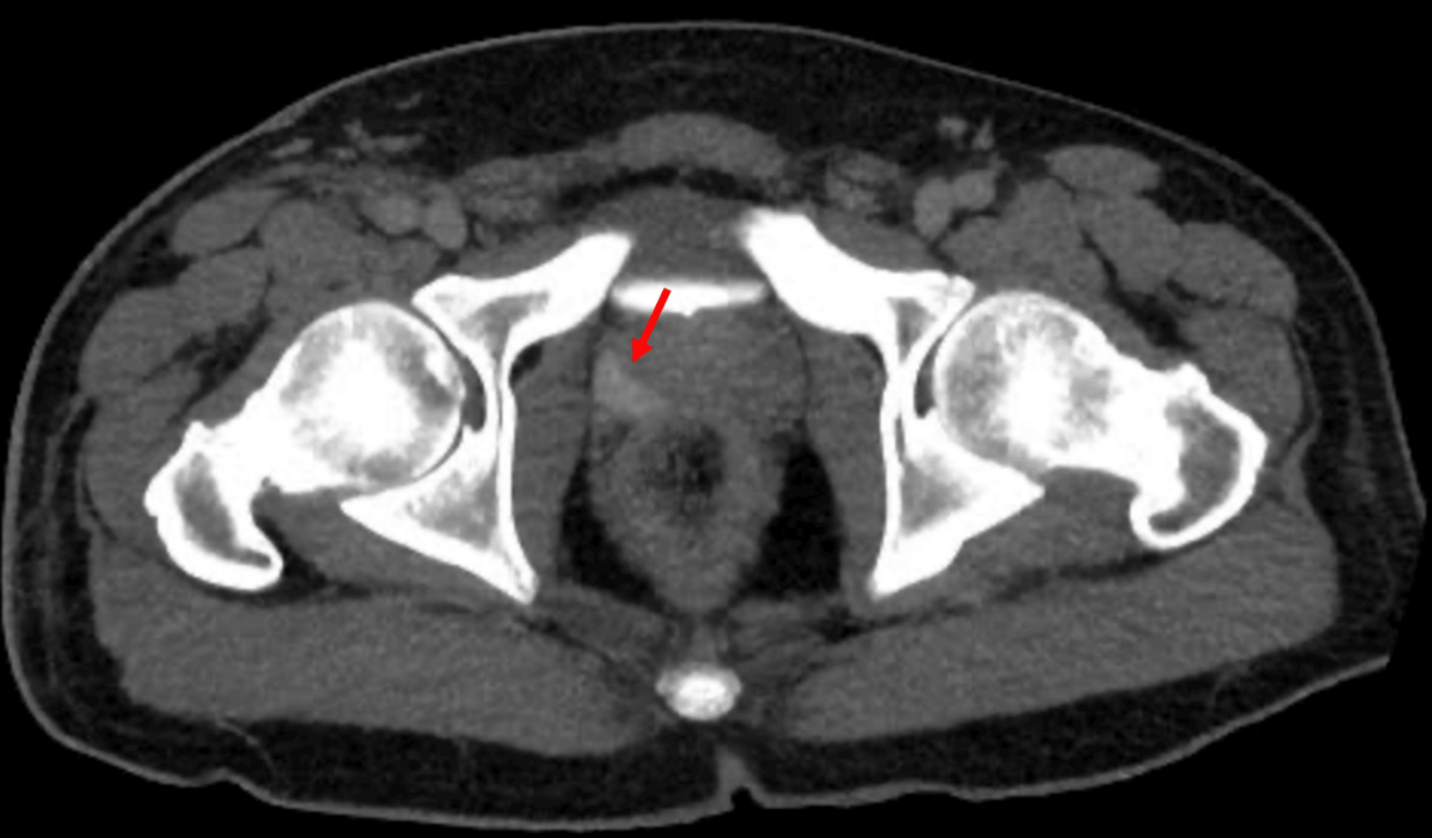
CT of the abdomen and pelvis. The red arrow points to the right-sided peripheral zone hyperdensity in the prostate.

Further imaging studies included a duplex ultrasound of the lower extremities, which showed no evidence of thrombosis, and an ultrasound of the upper extremities, which revealed acute partial thrombosis of the right basilic vein. A CT of the abdomen and pelvis revealed a prostate hyperdensity in the peripheral zone on the right, possibly due to an embolic event (Figure 4).

Laboratory tests were significant for a positive lupus anticoagulant panel and elevated Protein C activity, while Factor V activity was within normal limits (Table 1).

**Table 1:** Laboratory values. aPTT, activated partial thromboplastin time; INR, International Normalized Ratio

Test	Result	Reference range	Units	Interpretation
Lupus anticoagulant panel	Positive	Negative	N/A	Abnormal
Protein C activity	150%	70-140	%	High
Factor V activity	0.65 IU/dL	0.50-1.50	IU/dL	Normal
D-Dimer	1	<0.4	u/mL	High
INR	1.1	0.8-1.2	Ratio	Normal
aPTT	25	21-35	Seconds	Normal
Hemoglobin	14.2	Mean: 13.5-17.5	g/dL	Normal
Platelet count	160,000	150,000-400,000	mm^3^	Normal

These findings, in conjunction with the patient's history of cancer and multiple thrombotic events, raised suspicion for an acquired thrombophilia, possibly APS.

Management of this complex case presented significant challenges. The acute ischemic stroke due to LP2 occlusion was initially treated with tPA and thrombectomy, but the subsequent development of intracranial hemorrhages complicated further antithrombotic therapy. The discovery of a large PE required careful consideration of anticoagulation, balancing the need to treat the PE against the risk of expansion of the intracranial hemorrhages.

## Discussion

Pathophysiological mechanisms 

This case presents a compelling example of the synergistic effect between cancer-associated thrombosis and APS on thrombotic risk. These two distinct conditions combine to create a particularly hazardous thrombogenic environment [5]. The patient's history of duodenal cancer, despite being in remission for four years, likely contributed to the heightened thrombotic risk through several mechanisms.

Gastrointestinal cancers, including duodenal cancer as in our patient, are particularly associated with increased thrombotic risk due to their high tissue factor expression (expressed on tumor cells, tumor-associated macrophages, and released via TF-positive microparticles) and mucin production. Mucins can interact with selectins and trigger microthrombi formation, activating platelets and promoting their aggregation [6]. The location of the primary tumor also influences thrombotic risk, with gastrointestinal and pancreatic cancers carrying some of the highest risks among solid tumors.

Even in remission, several factors contribute to ongoing hypercoagulability. These include circulating tumor-derived extracellular vesicles, which can persist after apparent remission and carry procoagulant proteins and tissue factors. Furthermore, cancer-induced changes to the endothelial surface and persistent inflammatory states can continue to promote thrombosis long after the initial cancer treatment [7,8]. The risk is particularly significant in the first year after cancer diagnosis but can extend well into remission, as demonstrated in our patient.

The presence of lupus anticoagulant, characteristic of APS, further compounds this already complex prothrombotic state. While lupus anticoagulant interferes with phospholipid-dependent coagulation tests in laboratory settings, it paradoxically promotes thrombosis in vivo through disruption of anticoagulant functions of phospholipid-binding proteins, activation of endothelial cells, and induction of tissue factor expression [3,9].

The interaction between cancer-associated hypercoagulability and APS creates unique challenges. Cancer can induce the production of antiphospholipid antibodies, though their clinical significance may differ from traditional APS antibodies. Additionally, both conditions can affect the endothelial surface and promote tissue factor expression, potentially creating a synergistic prothrombotic effect. This combination may explain our patient's multiple thrombotic events despite being in cancer remission.

Diagnostic approach and challenges

The diagnostic workup in this case followed a systematic approach, driven by the patient's complex presentation with multiple thrombotic events. The initial evaluation began with neuroimaging studies, starting with a negative non-contrast CT, followed by a crucial CT angiogram that revealed occlusion of the LP2 segment. This guided the acute intervention decision and highlighted the importance of thorough vascular imaging in high-risk patients.

Laboratory studies revealed several significant findings: a positive lupus anticoagulant panel, elevated Protein C activity (150%), and normal Factor V activity (0.65 IU/dL). The D-dimer elevation (1 u/mL) and normal coagulation parameters (INR and aPTT) demonstrated that routine testing may not capture significant hypercoagulability. Notably, the normal aPTT despite positive lupus anticoagulant emphasizes that normal routine coagulation testing does not exclude significant coagulation abnormalities.

Comprehensive imaging proved essential for complete diagnosis. Beyond the initial brain imaging, chest CT angiography revealed a left lower lobe PE, upper extremity imaging showed right basilic vein thrombosis, and abdominal/pelvic CT demonstrated prostate findings suggestive of embolic phenomena. This pattern of multiple thrombotic events in various vascular beds suggested a systemic prothrombotic state rather than local factors.

The diagnostic complexity centered particularly on interpreting the lupus anticoagulant positivity in the context of cancer history. While malignancy can induce antiphospholipid antibodies, the presence of both arterial and venous thromboses during cancer remission strongly suggested concurrent cancer-associated thrombosis and APS. This diagnostic picture directly informed the challenging management decisions that followed, particularly regarding anticoagulation strategies.

Management challenges and therapeutic implications

The management of cancer-associated thrombosis requires careful consideration of multiple factors, including cancer type, stage, and treatment status. Traditional risk assessment tools like the Khorana score, which considers cancer type, body mass index, blood counts, and other factors, may need modification when APS is present. In our patient, the history of gastrointestinal cancer, coupled with positive lupus anticoagulant, created a particularly high-risk scenario requiring careful anticoagulation strategies.

The decision regarding inferior vena cava (IVC) filter placement was particularly challenging in this case. While IVC filters are often considered in patients with acute thrombosis who have contraindications to anticoagulation, such as our patient's intracranial hemorrhages, several factors influenced the decision against filter placement. First, the patient's thrombotic events were not limited to the venous system but also involved arterial circulation, suggesting that an IVC filter alone would not provide comprehensive protection. Additionally, in patients with hypercoagulable states such as APS and cancer-associated thrombosis, IVC filters themselves can become niduses for thrombus formation [10]. Furthermore, the patient's upper extremity thrombosis suggested that the prothrombotic state was not limited to the lower venous system. The risks of filter placement, including filter thrombosis, migration, and the potential need for future retrieval, were considered to outweigh the potential benefits in this complex case.

Subsequent intracranial hemorrhages complicated the initial treatment of acute ischemic stroke with tPA and thrombectomy. This scenario highlights the delicate balance required in managing anticoagulation in patients with both cancer and APS. The discovery of a large PE further complicated management, though interventional radiology ruled against PE thrombectomy due to hemodynamic stability.

Current guidelines for cancer-associated thrombosis generally recommend low-molecular-weight heparin (LMWH) over direct oral anticoagulants (DOACs), particularly in gastrointestinal cancers [11]. However, the presence of APS may influence this choice, as some studies suggest that DOACs may be less effective in APS patients with multiple thrombotic events [12]. In our patient, the presence of intracranial hemorrhages necessitated a more conservative approach with prophylactic anticoagulation initially.

Outcome and follow-up 

The patient's hospital course spanned 24 days, marked by a gradual but steady improvement. The initial neurological deficits, including right-sided weakness (0/5), improved to 3/5 in the upper extremity and 4/5 in the lower extremity by discharge. Serial neuroimaging demonstrated stable intracranial hemorrhages with gradual resolution of the surrounding edema and no new thrombotic events.

Anticoagulation management followed a carefully titrated approach. After a 14-day hold on therapeutic anticoagulation due to intracranial hemorrhages, the patient was successfully transitioned to therapeutic LMWH. The choice of LMWH over DOACs was based on both the patient's history of gastrointestinal cancer and the presence of APS. The patient tolerated therapeutic anticoagulation without hemorrhagic complications.

At three months follow-up, the patient demonstrated further neurological improvement with right-sided strength of 4+/5 throughout and independent ambulation. Repeat imaging showed complete resolution of the intracranial hemorrhages and no new thrombotic events. The patient's lupus anticoagulant remained positive on repeated testing, confirming the diagnosis of APS. Oncologic surveillance, including CT imaging and tumor markers, showed no evidence of cancer recurrence.

Future directions and conclusions

Successful management of cancer-associated thrombosis with concurrent APS demands precise therapeutic decisions, particularly when navigating both thrombotic and hemorrhagic complications. In this case, patient recovery relied on systematic thrombophilia evaluation and a carefully coordinated anticoagulation strategy. 

Several key research priorities emerge from this case. Prospective trials comparing anticoagulation strategies (LMWH, DOACs, and warfarin) in patients with both cancer and APS are urgently needed. Studies should examine factors determining optimal anticoagulation duration after cancer remission in APS patients, particularly focusing on the predictive value of serial antiphospholipid antibody testing. Additionally, research into risk prediction tools that incorporate both cancer-specific factors and APS parameters could better guide management decisions and prevent complications.

The development of evidence-based management protocols remains crucial, particularly regarding the timing of anticoagulation in patients with hemorrhagic complications. Questions about IVC filter placement timing, prophylactic anticoagulation strategies, and transitions between different anticoagulation approaches need systematic investigation. Early detection protocols for both thrombotic and hemorrhagic complications could significantly improve patient outcomes.

Moving forward, addressing these knowledge gaps will improve care for this challenging patient population. Integration of biomarker analysis and precision medicine approaches may help develop treatment algorithms that better account for individual patient factors and risk profiles. The lessons from this case emphasize that successful management requires careful consideration of both thrombotic and hemorrhagic risks, guided by regular monitoring and systematic follow-up.

## Conclusions

This case highlights the heightened thrombotic risk in a patient with a history of duodenal cancer and APS. Despite cancer remission, residual cancer cells and lupus anticoagulants contributed to severe thrombotic events. Even after cancer remission, residual cancer cells can continue to contribute to a hypercoagulable state, highlighting the persistent thrombotic risk in such patients. The presence of lupus anticoagulant further exacerbates this risk, making management more challenging.

The case demonstrates the need for thorough diagnostic evaluations and individualized treatment strategies, balancing anticoagulation with the risk of hemorrhage. Future research should focus on refining anticoagulation protocols and exploring long-term outcomes for patients with these combined risk factors.
